# Guidelines for releasing a variant effect predictor

**DOI:** 10.1186/s13059-025-03572-z

**Published:** 2025-04-15

**Authors:** Benjamin J. Livesey, Mihaly Badonyi, Mafalda Dias, Jonathan Frazer, Sushant Kumar, Kresten Lindorff-Larsen, David M. McCandlish, Rose Orenbuch, Courtney A. Shearer, Lara Muffley, Julia Foreman, Andrew M. Glazer, Ben Lehner, Debora S. Marks, Frederick P. Roth, Alan F. Rubin, Lea M. Starita, Joseph A. Marsh

**Affiliations:** 1 MRC Human Genetics Unit, Institute of Genetics and Cancer, University of Edinburgh, Edinburgh, UK; 2 Centre for Genomic Regulation (CRG), The Barcelona Institute of Science and Technology, Barcelona, Spain; 3 Department of Medical Biophysics, University of Toronto, Toronto, ON Canada; 4 Department of Biology, Linderstrøm-Lang Centre for Protein Science, University of Copenhagen, Copenhagen, Denmark; 5 Simons Center for Quantitative Biology, Cold Spring Harbor Laboratory, Cold Spring Harbor, New York, NY USA; 6 Department of Systems Biology, Harvard Medical School, Boston, MA USA; 7 Department of Genome Sciences, University of Washingtonand the, Brotman Baty Institute for Precision Medicine , Seattle, WA USA; 8 European Molecular Biology Laboratory, European Bioinformatics Institute, Wellcome Genome Campus, Hinxton, Cambridge, UK; 9 Vanderbilt University Medical Center, Nashville, TN USA; 10 Wellcome Sanger Institute, Cambridge, UK; 11 Broad Institute of MIT and Harvard, Boston, MA USA; 12 Department of Computational and Systems Biology, University of Pittsburgh School of Medicine, Pittsburgh, PA USA; 13 Bioinformatics Division, Walterand , Eliza Hall Institute of Medical Research, Parkville, Australia; 14 Princess Margaret Cancer Centre, University Health Network, Toronto, ON Canada; 15 Universitat Pompeu Fabra (UPF), Barcelona, Spain; 16 Institució Catalana de Recerca I Estudis Avançats (ICREA), Barcelona, Spain; 17 Department of Medical Biology, University of Melbourne, Parkville, Australia

## Abstract

Computational methods for assessing the likely impacts of mutations, known as variant effect predictors (VEPs), are widely used in the assessment and interpretation of human genetic variation, as well as in other applications like protein engineering. Many different VEPs have been released, and there is tremendous variability in their underlying algorithms, outputs, and the ways in which the methodologies and predictions are shared. This leads to considerable difficulties for users trying to navigate the selection and application of VEPs. Here, to address these issues, we provide guidelines and recommendations for the release of novel VEPs.

**Supplementary Information:**

The online version contains supplementary material available at 10.1186/s13059-025-03572-z.

## Background

Many different computational methods, known as variant effect predictors (VEPs), have been developed to assess the likely impacts of genetic variants [1–3]. These tools are often applied in the analysis and interpretation of human genetic variation, but also show considerable utility in other applications, such as evolutionary analyses [4, 5] and protein engineering [6, 7].

VEPs vary widely in their algorithms, training data, prediction interpretation, output format, and accessibility. Despite progress in the field, this diversity complicates end users’ ability to select the most suitable VEP and poses challenges for unbiased assessment, as new predictors often claim superiority over others [8]. Recent efforts have focused on independent benchmarking [9–12], but the sheer number of methods, their inconsistent naming (e.g., predictors of “variant effect,” “variant impact,” “functional effect,” “deleteriousness,” “pathogenicity,” or “mutational impact”), and the effort required to access predictions hinder identification and evaluation. Fair assessment also demands clear knowledge of training data, which is often poorly detailed in publications.

**Fig. 1:**
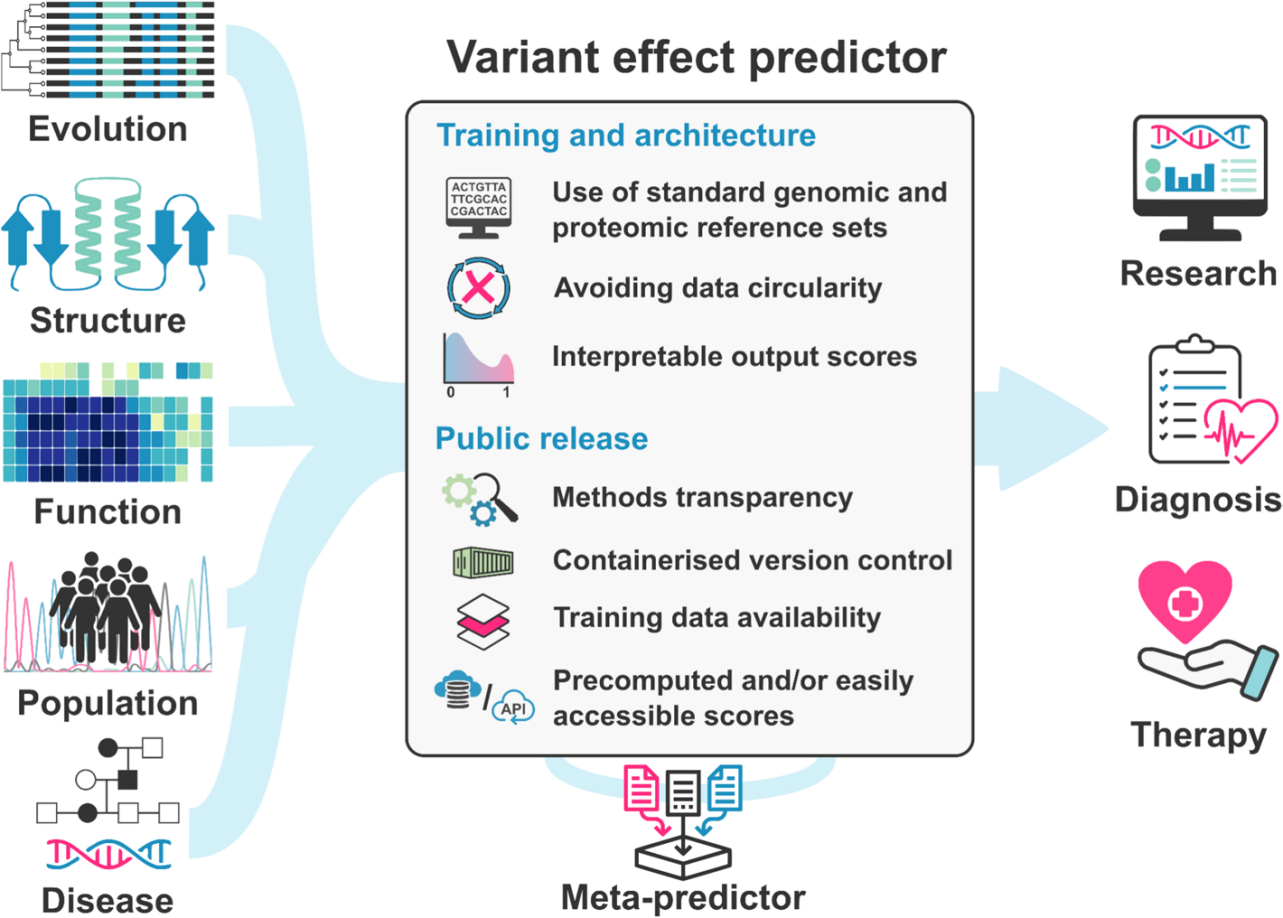
Overview of variant effect predictors, including common inputs and outputs, and guidelines for development and release

The Atlas of Variant Effects (AVE) Alliance coordinates researchers from around the world seeking to create comprehensive variant effect maps [13]. The AVE “*Analysis, Modeling, and Prediction*” workstream focuses specifically on computational methods for variant effect prediction and the analysis of multiplexed assays of variant effect (MAVE) data. Drawing on our experience with variant analysis tools, here we provide guidelines and recommendations that we believe should be considered when releasing a novel VEP, focusing primarily on tools that score pathogenicity or fitness (Fig. 1). However, most of our recommendations will remain applicable to tools for predicting other aspects of variant effects, like splicing [14], or changes in biophysical properties (e.g., protein stability [15], binding affinity [16], and aggregation propensity [17]). While some of our advice is specific to predictors of protein variant effects, we also discuss issues relating to nucleotide-level and non-coding predictors.

We hope that these guidelines will improve the evaluation of novel methods and facilitate their seamless incorporation into existing analysis pipelines. Furthermore, we believe that this will contribute to the broader adoption and utilization of VEPs within the scientific community, thereby accelerating our understanding of genetics and improving patient care. Ultimately, our goal is to support the creation of tools that are both scientifically rigorous and widely accessible, paving the way for advances in personalized medicine and genetic research.

## Results

### Sharing of methods and code

While many users focus solely on VEP outputs (variant effect scores), making the methods themselves available is essential. This allows novel variants to be tested and for methods to be more comprehensively evaluated. We strongly suggest that variant effect prediction methods be made freely available and open source, with a clear Open Source Initiative (https://opensource.org) approved license. By making VEP methods and their corresponding codebases accessible and clearly documented, developers empower researchers across the globe to contribute to the evolution of these tools, enhancing their accuracy, efficiency, and utility. Importantly, this includes not only the final trained models but also the data pre-processing scripts, ensuring full transparency in dataset construction and model development. Providing these resources enables precise replication of results, facilitates improvements to existing methods, and avoids issues where key methodological details remain undisclosed. Sharing pre-processing scripts is also critical for detecting and preventing data leakage, for example, through unintentional duplication, use of proxy labels, or inclusion of information not available at prediction time. Finally, making code available could enable the calculation of scores using, for example, different reference genomes or trained using variants from different populations, with potential importance in health equity [18, 19].

In addition to code, providing the names and values of the input features used in predictions would significantly enhance transparency. Where applicable, authors should also report feature importance, for example, by assessing the impact of feature randomization on model performance, as extracting meaningful relative weights from complex models such as neural networks is typically not feasible. This approach could also reduce the risk of double counting evidence when applying ACMG/AMP guidelines for variant classification.

In the past, many VEP methodologies have been made available as web servers, through which individual variants can be queried. While this can be convenient for end users who are interested in small numbers of variants, making a method available only as a web server severely limits the potential for a method to be independently assessed. At the very least, web servers should offer an application programming interface (API) for bulk queries, if a pre-calculated download is not an option. Concerningly, we have found many examples of such online predictors disappearing from the web after only a few years, undermining their long-term utility.

Hosting the code for a VEP on a public, open-source platform like GitHub (https://github.com) or Huggingface (https://huggingface.co) provides high levels of visibility, version control, and the opportunity to integrate documentation. Repositories such as Kipoi [20] are also useful for depositing models, facilitating broader access to the necessary tools for exact replication of model predictions. Releasing models with their trained parameters is crucial for reproducibility and utility. This practice addresses the inherent stochasticity in training machine learning models, ensuring that the reproducibility of a VEP is not compromised. Releases should also be stably archived, ensuring reproducibility even when the model is updated. A containerized version of the method, utilizing platforms such as Docker (https://www.docker.com) or Apptainer (formerly known as Singularity, https://apptainer.org), can also be very useful, especially for cross-platform analysis or where installation poses challenges. These tools encapsulate the method and its dependencies in a container, ensuring that it can be run seamlessly across different computing environments.

It is also important to clearly document the methodology underlying a novel VEP. This should include a list of all the features included in the final model with links to sources and code or replicable methodology that can be used to engineer these features if necessary. For methods utilizing macromolecular structures, the source of these should be clearly identified. Ideally, whenever licensing permits, providing direct access to source files ensures reliability and reproducibility by avoiding dependence on external databases.

In addition to making VEP methodologies and resources transparently available, it is helpful to communicate the computational cost and runtime associated with these tools. This is particularly relevant for GPU-based models, where memory constraints may limit scalability. Developers should report memory usage and inference time, and where possible, indicate how performance scales with protein length (e.g., using Big O notation) to help users assess feasibility on their available hardware. A VEP capable of running genome-wide analyses on a standard laptop offers different possibilities compared to one requiring substantial computational resources for only a few protein assessments. This distinction not only impacts the practicality of the tool for various research applications, but also raises important considerations regarding energy consumption and sustainability [21].

### Interpretability of variant effect scores

The outputs produced by different VEPs can vary widely. For tools that predict effects on specific biophysical properties like stability or interactions, the meaning of the outputs is often very clear (e.g., predicted ΔΔG in units of kcal/mol). However, most VEPs provide a variant effect score that may be interpreted as being related to the likelihood of a given variant being pathogenic, or damaging to function or fitness. Importantly, variant effect scores very rarely indicate whether a variant disrupts or enhances a function [22], which can have clinical implications in genes where loss-of-function and gain-of-function variants cause different diseases [23], and because gain-of-function variants tend to be predicted less well by most VEPs [24]. Therefore, we encourage future methods to focus more on predicting mechanisms and inheritance.

The interpretation of variant effect scores is often difficult and the scales can vary widely. The most common scale ranges from zero, least damaging, to one, most damaging. However, we note that this directionality is opposite to what is commonly used for the outputs of MAVE experiments, in which a value of one often represents wild-type fitness and zero corresponds to the fitness of a null (e.g., nonsense) variant [25]. While, ideally, VEPs and MAVEs would be calibrated to similar scales, we suggest that creators of new VEPs consider adopting zero-to-one scales of least-to-most damaging, so long as this does not obfuscate the interpretation of the variant effect score. This matches the most common convention and aligns with the directionality used by the large majority of current methods.

It is important to include an explanation of how scores can be compared. For many methods, variant effect scores can be compared across genes (e.g., two different variants with the same score from two different genes would be considered equivalent in terms of their likelihood of being pathogenic). However, for others, the scales are defined at the level of individual genes, and scores for variants from different genes are not necessarily comparable. For example, DeepSequence models are generated on a per-protein basis, with the scores representing the likelihood ratio between mutant and wild-type residues [26]; thus, scores from different proteins are not directly comparable.

Some methods provide labels along with variant effect scores. These are often desired by end users, but also come with a risk of overinterpretation. The rationale and thresholds must be clearly explained and justified, and care should be taken about the choice of labels. For example, AlphaMissense classifies many possible human variants as “*likely pathogenic*” and “*likely benign*” [27]. However, there already exist very clear clinical definitions of these terms that are completely distinct from the definitions used by AlphaMissense [28, 29]. This has considerable potential to confuse end users, who may include patients or patient families. If labels are to be provided alongside variant effect scores, we suggest that terms that are distinct from the clinical classifications be used. For example, the widely used PolyPhen- 2 predictor defines thresholds for “*possibly damaging*” and “*probably damaging*” [30]; these terms should have a much lower chance of confusion with the well-established clinical classifications. An alternative could be to use the Sequence Ontology terms “*functional_normal*” and “*functionally_abnormal*” [31], which could be particularly relevant as more mechanism-centric predictive methods are introduced.

One emerging strategy for facilitating the use of VEP scores as evidence in clinical variant interpretation is through calibration to ACMG evidence strength levels [32, 33]. Importantly, however, even after using a well-validated calibration, references to pathogenicity should only describe scores as *evidence towards* pathogenicity or benignity, rather than defining variants as such.

### Accessibility of predictions

The success of a VEP is intricately linked to the availability of its outputs. The free and unrestricted availability of these scores is essential for the method to be widely used. Ensuring that these data are not only available but Findable, Accessible, Interoperable, and Reusable aligns with the FAIR Guiding Principles for scientific data management [34]. Adhering to FAIR principles in disseminating variant effect scores facilitates broader participation in genomic research, enhances the reproducibility of scientific findings, and accelerates the translation of genomic data into actionable clinical insights.

Unfortunately, certain methods impose restrictive licensing terms on their predictions, hindering independent performance assessments and, consequently, limiting user confidence and impeding integration into clinical variant assessment frameworks. We therefore advocate for freely available data to enable scientific discovery and clinical decision-making. The argument has been made against making variant effect scores freely available to avoid their incorporation into other predictors and thus confounding performance assessment [35]. While there are potential complications arising from such approaches, we believe that the issue of restricted data preventing the very assessments needed to address potential confounding effects is far more concerning, and that such closed methods can never receive the open, independent assessments needed to be considered trustworthy by the community.

The methodology behind a VEP dictates the most appropriate format for sharing its predictions. For many currently available VEPs, predictions are performed at the protein level. Thus, scores should be provided with respect to the appropriate reference sequence against which the prediction was performed. In our experience, most protein-level VEPs output predictions using canonical UniProt protein sequences. Going forward, we recommend that developers utilize transcripts recommended by the Matched Annotation from NCBI and EMBL-EBI (MANE) collaboration [36]. The MANE Select transcript set includes a default recommended transcript for nearly all protein coding genes and matches the UniProt canonical isoform in the vast majority of cases. In addition, the MANE Plus Clinical transcripts are defined for the relatively small number of genes where a single transcript is not sufficient to report all clinically relevant variants. Therefore, we suggest that, for protein-centric methods, variant effect scores ideally be provided for all possible single amino acid substitutions across all protein sequences corresponding to MANE Select and MANE Plus Clinical transcripts. However, we recognize that this is not always computationally feasible. In these cases, we suggest that predictions be provided for as many human proteins as possible, focusing on those of greatest clinical relevance (e.g., genes included in the Gene Curation Coalition database [37] or the ACMG secondary findings list [38, 39]), and those for which MAVE datasets have been published, enabling MAVE-based benchmarking.

Other VEPs make predictions at the nucleotide level. The further advancement of such methods is critical to interpreting the vast majority of human genetic variation that occurs in non-coding regions [40]. For methods that make predictions outside of exonic regions, variant effect scores should be shared using genomic coordinates based on a specific, versioned reference genome assembly.

In some cases, protein-based methods have their predictions shared in terms of genomic coordinates. While this has some advantages in terms of incorporation into genomic analysis pipelines, we suggest that, if predictions are made at the protein level, then predictions should also be provided at the level of the same protein sequences. In addition, most single amino acid substitutions cannot be achieved by single nucleotide changes, thus losing some information if only nucleotide-level predictions are provided. While this has no impact on analyses of single nucleotide variants, there are many examples of pathogenic single amino acid substitutions caused by multi-nucleotide changes. These substitutions may also be of interest for other reasons, such as comparison to MAVEs or for protein engineering applications. Separate tools, such as the Ensembl Variant Effect Predictor [41], or the Ensembl REST API [42] and EMBL-EBI Proteins API [43], can be used to map protein-level variants to genomic coordinates, if necessary. Additionally, tools like ProtVar provide dedicated functionality for mapping genomic variants in coding regions directly to their corresponding changes in the UniProt primary isoform [44]. When predictions are performed or provided at the nucleotide level, but analyses are at the protein level, there may be ambiguity when different variant effect scores are provided for different single nucleotide variants that translate into the same amino acid change. We suggest reporting the most deleterious score, in addition to also sharing the nucleotide-level predictions.

Some VEPs are able to make predictions for variants other than single amino acid or single nucleotide substitutions. At the protein level, it may be possible to provide comprehensive predictions across the human proteome for truncations and for single amino acid insertions and deletions. However, it would be unrealistic to provide predictions for all possible variants when considering larger sequence changes involving indels and multi-amino acid substitutions. Similarly, for nucleotide-level predictors, it may be infeasible to provide complete predictions for anything other than single nucleotide variants for a limited subset of the genome. In these cases, the availability of the method for users to run specific predictions of interest is absolutely essential. In addition, predictions could be specifically provided for larger sequence variants known to be pathogenic [45] or present in the human population [46].

When sharing variant effect scores for single amino acid substitutions mapped to a clearly defined reference sequence (e.g., a MANE transcript or UniProt ID), a simple format like “P316D” may suffice for convenience in computational contexts. However, we strongly recommend using the Human Genome Variation Society (HGVS) notation [47] (e.g., p.Pro316 Asp) as the standard, as it minimizes ambiguity—particularly in multi-gene or clinical settings where isoforms, numbering discrepancies, or nucleotide-level confusion could arise—and better supports complex variants like indels. For larger and more complex variants, we recommend considering the Global Alliance for Genomics and Health (GA4GH) Variation Representation Specification (VRS) [48]. Additionally, providing ClinGen Allele Registry IDs [49] alongside these notations can further enhance variant identification and interoperability with clinical and research databases.

Although most of the current interest in VEPs is focused on human genetic variation, and many VEPs have been developed that only provide predictions for human variants, some VEPs, particularly those based on unsupervised learning approaches, are applicable to variants from any species. While it is clearly not realistic to provide predictions for all variants across all species, we suggest that, in addition to predictions across the human proteome, variant effect scores be provided for any variants present in MaveDB [50] and/or ProteinGym [51] to facilitate independent benchmarking and analysis.

For sharing variant effect scores and other essential data, we strongly recommend deposition in a well-established public repository that provides a DOI for reference, such as Zenodo (https://zenodo.org), Dryad (https://datadryad.org), or the Open Science Framework (https://osf.io), rather than hosting them on the authors’ website. This practice not only ensures the long-term availability and utility of the data but also helps its distribution, since many of these repositories have an API that allows fast programmatic access to data.

### Availability of training data

Most VEPs that have been developed to date are based on supervised learning strategies based on training against labeled datasets of variants, usually split into pathogenic and benign, sourced from databases like ClinVar [45] and gnomAD [46]. A critical issue in the field of variant effect prediction is that of data circularity, whereby the performance of VEPs is evaluated using either variants that were directly or indirectly used in training, thus inflating apparent performance [52]. Therefore, the performance of different VEPs is heavily influenced by the test datasets, and many tools perform markedly worse when applied to novel missense variants [10]. This includes biases in databases like ClinVar, where labels may be influenced by predictions from existing VEPs, further complicating fair evaluation [52].

To address this problem, recent studies have used correlations with independent MAVE datasets to compare VEP performance [9, 11, 51]. While this can be useful to compare different VEPs, it is worth noting that MAVEs do not always probe functions that are central to the development of disease or use a disease-relevant tissue context. If a more traditional assessment of discrimination between pathogenic and benign variants is desired, it is essential to ensure that none of the variants used in VEP training, or other variants at the same positions, is present in the evaluation set, to avoid confounding from type 1 (variant-level) circularity [52, 53]. Moreover, given the issues associated with type 2 (gene-level) circularity, where a model trained on variants from specific genes may exhibit inflated performance by leveraging learned associations between those genes and pathogenicity when tested on different variants from the same or homologous genes, it would be safest to exclude from evaluation any variants from genes used in training of the VEP, or even genes homologous to any genes used in training. Alternatively, type 2 circularity can be avoided by assessing performance only at the level of individual genes [53], or by using the same balance between pathogenic and benign variants across all genes in the test set [54].

Given these issues, it is crucial for the integrity and transparency of a VEP that all variants employed in its training are disclosed upon release. Ideally, these should be shared in the same format as the variant effect scores themselves, rather than merely referencing the databases, due to the dynamic nature of these resources and the potential variability in mapping methods to different sequence identifiers. In situations where controlled access datasets are used and a comprehensive list of training variants cannot be openly shared, it becomes imperative to explicitly detail the version of the dataset, along with the processing and filtering methods applied. This ensures that, despite the restrictions, the original training set can be accurately reconstructed by others. For this reason, we strongly advise against using any private or commercial datasets for training if the variants cannot be fully disclosed.

Difficulties associated with circularity can become particularly acute with ensemble or meta-predictors, which use the outputs of other VEPs as features in their training. If other supervised models are used as features, then the identities of the variants used to train those models are required for fair assessment.

Some VEPs have been released that do not train on pathogenic variants, but do contain information on the allele frequencies of variants present in the human population, or their frequencies in primate species [27, 55]. It is essential that the identities of these variants or their mapped human variants be provided. We emphasize that such VEPs face the same issues of circularity in performance evaluation as other supervised VEPs. In particular, allele frequency is very commonly used as evidence in the classification of variants as benign [29]. Thus, when assessing discrimination between pathogenic and benign variants, a VEP that is trained or tuned using allele frequencies will have effectively been exposed to much of the benign dataset, which can inflate apparent performance [56].

An important application of VEPs is in the interpretation of extremely rare variants. As it has been shown that common benign variants are not representative of rare benign variants [57, 58], users may wish to choose VEPs that perform well on test sets of exclusively rare variants. Hence, those training VEPs may wish to consider excluding common benign variants from their training sets or downweighting their influence.

Some VEPs now use MAVE data in their training [58–60]. This introduces new circularity issues and can confound MAVE-based benchmarking if the datasets used for training are not excluded. As long as the MAVE datasets are present in MaveDB or a benchmark such as ProteinGym, it should be sufficient to cite their accession if used in training. In the event that MAVE data are hosted at a location that may become unavailable (e.g., on a group’s website), then all variants used for training should be provided, similarly to database-sourced training variants.

There are unique issues associated with VEPs that work on the nucleotide level and are focused on predicting non-coding variant effects. It is crucial for these models to specify the resolution used in training, the genomic regions used (e.g., whole genome, promoters, or UTRs), and the molecular/evolutionary modalities considered. These details directly influence how the effects of variants are interpreted and delineate the scope of sequences for which the model can accurately provide predictions.

Increasingly, many VEPs are based upon unsupervised approaches, often taking multiple sequence alignments as input [61, 62]. Although it has not been common practice in the past, we suggest that it is important to make the sequence alignments underlying these models available, along with careful documentation of how the alignments were generated. This would allow assessment of the extent to which the alignment depth and quality influence prediction performance. Furthermore, non-human variants, especially from primates, have occasionally been used as “benign” variants for VEP evaluation. This could lead to another level of circularity, if these non-human species have been included in the sequence alignment. Thus, the availability of sequence alignments and knowledge of the species on which the model is based can be crucial.

The other increasingly popular unsupervised approach, protein language models, are trained directly on protein sequence information and do not require alignment generation for prediction [63]. While the identity of the databases used to train such models is often provided, model-specific clustering and filtering procedures can obfuscate the exact sequences that were used during training. We suggest that authors of language models and similar methods provide both the database version and all sequence identifiers that went into training the final version of the model.

### A list of currently available variant effect predictors

To increase the visibility and discoverability of new VEPs, we have compiled an extensive list of tools at https://www.varianteffect.org/veps. This includes classifications in terms of their underlying methodologies and features. We also include details on the author-recommended pathogenicity prediction thresholds and links to their web servers, variant effect scores, training data, and code downloads. A current snapshot of our VEP list is provided as Table S1. While this list is not yet fully comprehensive, given the huge number of tools that have been published, we are actively adding new methods as we identify them, and we strongly encourage submissions of new methods to be included, or updates of old methods, using the web form available at that site. We also recommend the Variant Impact Predictors Database (VIPdb) as a resource for discovering VEPs and related tools [64].

## Conclusions

The guidelines presented here aim to streamline VEP development, sharing, and evaluation by tackling data availability, interpretability, transparency, and circularity. Advocating for freely shared variant effect scores, open-source methods and code, and strict training data standards, we seek to boost VEP reliability, usability, and integrity. Promoting best practices in sharing predictions and methodologies aids independent assessment, clinical integration, collaboration, and innovation. As VEPs advance, they will likely gain greater weight in clinical variant interpretation, either alone [33] or in combination with increasingly available MAVE data [65]. Adhering to these guidelines will enhance personalized medicine and genetic disease understanding, aligning with calls for standardized, rigorous VEP practices in genomic medicine [22].

## Supplementary Information

Supplementary Material 1.

## Data Availability

No datasets were generated or analysed during the current study.
